# Lipid droplets of protozoan parasites: survival and pathogenicity

**DOI:** 10.1590/0074-02760210270

**Published:** 2022-02-16

**Authors:** Victor de Souza Tavares, Monara Viera de Castro, Rayane da Silva Oliveira Souza, Iana Kátia Araújo Gonçalves, Jonilson Berlink Lima, Valéria de Matos Borges, Théo Araújo-Santos

**Affiliations:** 1 Universidade Federal do Oeste da Bahia, Centro das Ciências Biológicas e da Saúde, Núcleo de Estudos de Agentes Infecciosos e Vetores, Barreiras, BA, Brasil; 2 Fundação Oswaldo Cruz-Fiocruz, Instituto Gonçalo Moniz, Laboratório de Inflamação e Biomarcadores, Salvador, BA, Brasil

**Keywords:** lipid droplet, lipid bodies, protozoan, parasites, oxidative stress, lipid metabolism, eicosanoid

## Abstract

Lipid droplets (LDs; lipid bodies) are intracellular sites of lipid storage and metabolism present in all cell types. Eukaryotic LDs are involved in eicosanoid production during several inflammatory conditions, including infection by protozoan parasites. In parasites, LDs play a role in the acquisition of cholesterol and other neutral lipids from the host. The number of LDs increases during parasite differentiation, and the biogenesis of these organelles use specific signaling pathways involving protein kinases. In addition, LDs are important in cellular protection against lipotoxicity. Recently, these organelles have been implicated in eicosanoid and specialised lipid metabolism. In this article, we revise the main functions of protozoan parasite LDs and discuss future directions in the comprehension of these organelles in the context of pathogen virulence.

Biogenesis of lipid droplets in protozoan parasites

Most cells present lipid droplets (LDs), also called lipid bodies, which are cytoplasmic organelles involved in lipid compartmentalisation and signaling. All LDs, regardless of cell type, have a similar molecular composition: a hydrophobic neutral lipid core coated by a monolayer of phospholipids.^1^ Despite the poorly understood cellular and molecular mechanisms of LD biogenesis in protozoan parasites, knowledge of mammalian cells can shed light on cellular mechanisms and protein involvement in LD formation.^2^ More than just an accumulation of neutral lipids, the majority of eukaryotic cells LDs remain stable due to structural proteins such as perilipin, adipose differentiation-related protein (ADRP) and tail-interacting protein of 47kDa (TIP47). These comprise PAT proteins; they support fatty acid (FA), triacylglycerol (TAG) and cholesteryl ester (CE) uptake and are crucial to LD formation de novo in mammalian cells.^2^ ^,^ ^3^ ^,^ ^4^ ^,^ ^5^ Although none of the genes related to PAT protein production have been described in parasites genomes, the LD kinase (LDK) found in trypanosomatids is the only protein in parasitic protozoa known to be responsible for performing a similar function.^6^ Moreover, other organelles take part from the origin until the LD turnover.^7^ The endoplasmic reticulum (ER) provides the structural molecular components necessary for biogenesis, and mitochondria capture the molecules mobilised in LDs for use in metabolic functions^8^ (Figure and Table I).

**Figure:**
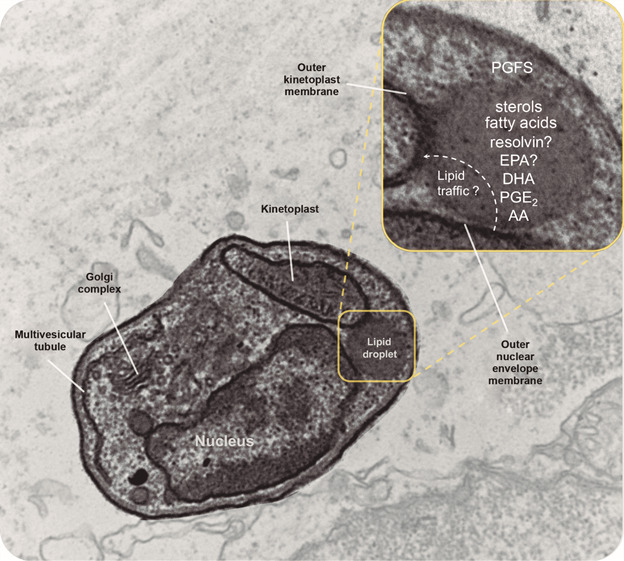
Schematic representation of interaction between lipid droplet (LD) and protozoan organelles. Schematic drawing on transmission electron microscopy illustrating the interaction lipid droplets, outer nuclear envelop membrane, and kinetoplast. Lipid droplet is responsible to storage and metabolise cholesteryl ester, fatty acids,^10^ ^,^ ^24^ ^,^ ^27^ and lipid mediators and their precursors, such as PGE_2_,^24^ but the presence of specialised lipids such as resolvins, and their precursors are still uncertain. In addition, LDs participate from the lipid traffic between intracellular organelles. Note the proximity of the outer nuclear envelop membrane and outer kinetoplast membranes to the LD hemi-membrane. TEM from *Leishmania* amastigote. *Toxoplasma gondii* and *Trypanosoma cruzi* also present LD interactions with mitochondria and endoplasmic reticulum.^10^ ^,^ ^15^

**TABLE I:** Functions and presumable composition of lipid droplets in the protozoa parasites

Species	Roles of LD	Components	References
*Leishmania infantum*	Virulence Eicosanoid metabolism Lipid traffic and organelle membrane interactions	Prostaglandin F2 α synthase, and Arachidonic Acid*	Araújo-Santos et al.^27^
*Leishmania amazonensis*	Increase during cell death induced by oxidative stress	Undetermined	da Silva Rodrigues et al.^19^
*Toxoplasma gondii*	Cholesterol, DAG, and TAG metabolism Storage of neutral lipids	Cholesterol, TAG, and DAG molecules TgACAT1, TgACAT2, TgDGAT, and Tglipin enzymes^#^	Quittnat et al.^10^ Nishikaea et al.^11^ Lige et al.^12^
*Trypanosoma brucei*	Growth and differentiation TAG and phospholipids metabolism	TAG; Phospholipids; LDK; TbLpn enzyme^#^	Flaslohper et al.^6^ Dawoody et al.^17^
*Trypanosoma cruzi*	Lipid traffic, storage, and metabolism Organelle communications Eicosanoid production	Neutral lipids, sterols, cholesterol, cholesteryl esters, acylglycerols, phospholipids, fatty acids, arachidonic acid, and PGE_2_	Toledo et al.^24^ Pereira et al.^15^
*Plasmodium falciparum*	Replication and membrane generation of the parasites Heme detoxification effects Induces β-hematin formation in the digestive vesicles.	Neutral lipids and PfDGAT enzyme^#^	Vielemeyer et al.^9^ Jackson et al.^30^ Ambele et al.^32^

*: localisation in lipid droplets was not demonstrated; ^#^: localisation in endoplasmic reticulum. Enzymes involved in the biogenesis of lipid droplets; TAG: triacylglycerol; Tg: *Toxoplasma gondii*; Pf: *Plasmodium falciparum*; ACAT: Acyl-CoA cholesterol acyltransferase; DGAT: Diacyl-CoA:cholesterol acyltransferase; LDK: lipid droplet kinase; COX: cyclooxygenase; PGFS: prostaglandin F_2α_ synthase; PGE_2_: prostaglandin E_2_; TbLpn: *Trypanosoma brucei* Lipin.

Several protozoan pathogens take advantage of lipid metabolite sequestration in the host. *Toxoplasma gondii* and *Plasmodium falciparum* display several proteins related to the accumulation of CEs in LDs.^9^ ^,^ ^10^ ^,^ ^11^ ^,^ ^12^ ^,^ ^13^ Acyl-CoA: cholesterol acyltransferase (ACAT)-related enzymes TgACAT and TgACAT2, and acyl-CoA: diacylglycerol acyltransferase (DGAT)-related enzyme TgDGAT, which are responsible for metabolising and synthesising CEs and TAGs contributing to lipid storage in the LDs of these parasites.^9^ ^,^ ^10^ ^,^ ^11^ ^,^ ^12^ ^,^ ^13^ Moreover, *T. gondii* genome encodes a single lipin, *TgLIPIN*, which is responsible for converting phosphatidic acid to diacylglycerol and phosphate.^14^ *T. gondii* knockouts of *TgDGAT* and *TgLIPIN* decrease LD formation.^10^ ^,^ ^14^ suggesting that these genes play a central role in LD biogenesis in this parasite. The physical proximity of protozoan LDs to the ER and mitochondria,^15^ suggests that LD biogenesis is both functionally and morphologically dependent on intracellular organelle interactions. Especially during nutrient starvation, the trafficking of FAs from the LDs to into the mitochondria protects cells against the lipotoxicity of autophagic processes.^14^ ^,^ ^16^ The depletion of the *Trypanosoma brucei* lipin homolog (*TbLpn*) decreases the number of LDs, simultaneously affecting the structural integrity of the mitochondria and dramatically reducing ATP production,^17^ suggesting a role of protozoan LDs in lipid traffic between the ER and mitochondria in parasites (Figure and Table I).

In trypanosomatids, a protein kinase localised intracellularly in close association with the ER and the LD hemi-membrane surface produced insights into the mechanisms responsible for signaling pathways of LD biogenesis in protozoans. Named lipid droplet kinase (LDK) due to a protein kinase domain and LD association, this protein is critical for LD formation. LDKs are activated by autophosphorylation, and induce LD biogenesis in *T. brucei*.^6^ More studies are necessary to elucidate kinases and signaling pathways involved in LD biogenesis in other protozoan pathogens.

The cell death process also triggers the production of LDs in parasites.^18^ Antiparasitic drugs act by interfering with lipid metabolism or inhibiting mitochondrial activity, concomitantly inducing LD formation in trypanosomatids.^19^ Treatment with amiodarone in *Leishmania amazonensis* was found to lead to degenerative changes mainly in the structure, membrane, and function of mitochondria. This resulted in cell death marked by a dose-dependent accumulation of LDs, which were near autophagosomes and multivesicular bodies suggesting the induction of LD biogenesis as a result of neutral lipid storage from membrane degradation.^20^

Cells infected by protozoan parasites have their lipid metabolism and LD production altered in response to parasite-derived stimuli. When infecting hosts, parasites such as *T. gondii* and *Leishmania major* increase lipid accumulation inside the LDs of host cells and employ them in their own metabolism.^11^ ^,^ ^21^ ^,^ ^22^ Further, *T. gondii* can interfere in the distribution of host organelles, such as mitochondria and LDs, by attracting them to parasitophorous vacuoles to access the lipids and incorporate them into their own LDs. Such strategies are vital for the survival inside the host, allowing them to recruit more resources for their lipid stores and metabolic processes.^13^ ^,^ ^23^ Furthermore, LDs of *Trypanosoma cruzi* trypomastigotes present large amounts of prostaglandin E2 (PGE_2_), an important immunomodulatory,^24^ showing that demands arising from inflammatory processes also trigger LD biogenesis, which likely supports the parasite’s survival during the course of infection.

Protozoan parasite LDs and lipid storage

LDs are dynamic organelles that interact with several other cellular organelles; they are the storage and synthesis site of lipids, including important inflammatory mediators, in pathogenic protozoa.^25^ ^,^ ^26^ Epimastigotes of *T. cruzi* have LDs predominantly composed of neutral lipids, mainly cholesterol esters and there are indications that these organelles function as storage sites for exogenous cholesterol, mainly in the form of cholesterol esters.^15^ In addition, *T. cruzi* LDs have acylglycerols and, among bioactive lipids, polyunsaturated fatty acids, such as arachidonic acid (AA) and docosahexaenoic acid (DHA),^24^ are part of the fraction of free FAs present in the LDs of this parasite^15^ (Figure).

The formation of LDs can be modulated by lipids exogenous to protozoa. In *Leishmania infantum* and *T. cruzi*, exogenous AA induces the formation of LDs, as well as the formation of eicosanoids.^24^ ^,^ ^27^ Stimulating procyclic forms of *T. brucei* with oleic acid (OA) induces an increase in the number of LDs, and therefore, the synthesis and storage of TAGs in LDs of the protozoan.^28^ Supplementation of unsaturated FAs in *T. gondii* cells results in extremely large LDs and impairs protozoan replication, while saturated FAs do not lead to these effects.^29^ Furthermore, toxoplasma is unable to use FAs as a source for ATP synthesis, and both exposure to exogenous unsaturated FAs and inhibition of TgDGAT lead to excess lipids in the cytoplasm and consequent lipotoxicity due to alteration of intracellular membranes and, ultimately, to the death of the protozoan.^29^

In some protozoans, the increase in LDs is related to the differentiation process. *Leishmania* presents an increase in the number of LDs during the process of metacyclogenesis and amastigogenesis, presenting more LDs in the infectious forms.^27^ *P. falciparum* mobilises lipids from LDs, serving as a source of precursors for the generation of membranes and replication of the protozoan.^30^ LDs of *P. falciparum* can be found by performing physical interactions with the *Plasmodium* food vacuole (VF).^30^ Since neutral lipids facilitate the formation of β-hematin,^31^ ^,^ ^32^ the physical interaction between VF and LD could be responsible for supplying these lipids to the parasite, so that hematin crystallisation occurs more efficiently. In fact, not only has the formation of hemozoin crystals on the surface of LDs been documented, but also the possibility that the diameter of the organelle controls the size of these crystals.^32^ Therefore, LD may serve as a place for the formation of β-hematin, but further studies are needed to test this hypothesis.

Lipid droplets protect protozoan parasites against oxidative stress

The increase in LDs is a defense mechanism of pathogens against external aggressions arising from the host´s immune system or from antiparasitic drugs. Some studies suggest that the change in the lipid profile of parasites may be a resistance mechanism, since strains of resistant parasites show changes in their lipid profiles when compared to susceptible strains.^33^ Therefore, LDs may be intrinsically related to the parasite´s defense mechanisms, as LDs are the sites of lipid synthesis and storage.

A factor closely related to the maintenance of parasitic life is mitochondrial activity, which can be altered by an imbalance in the production of free radicals, as well as other reactive species. Trypanosomatids present detoxification mechanisms of reactive oxygen species,^34^ ^,^ ^35^ ^,^ ^36^ ^,^ ^37^ ^,^ ^38^ which involves at least two enzymes from the superoxide anion degradation pathway - the superoxide dismutase 1^39^ and trypanothione reductase enzymes^37^ being the two best-described parasitic enzymes. Antiparasitic drugs usually cause mitochondrial damage leading to the death of the parasite with an intracellular accumulation of lipids in LDs.^19^ Clomipramine induces cell death by inducing oxidative stress, which occurs concurrently with an increase in the number of LDs and peroxided lipids in *L. amazonensis*. The effects of clomipramine are reversed in the presence of antioxidants, such as N-acetylcysteine (NAC), suggesting that the number of LDs increases in *L. amazonensis* to store peroxided lipids, protecting the cell from damage.^19^ A similar defense mechanism occurs during oxidative stress induced by miltefosine in strains of *Leishmania donovani*, in which drug-resistant parasites increase the expression of genes related to antioxidant mechanisms.^40^

In addition to the accumulation of oxidised lipids, LDs are the main sterol storage sites,^15^ ^,^ ^41^ and the production of steroids can be a defense mechanism of protozoa, such as *L. donovani*, against oxidative stress generated by reactive species. Drugs with steroid biosynthesis inhibitory activity have an antiparasitic effect. However, inhibition of sterol synthesis alone is not able to suppress *L. donovani* intracellular survival.^42^ The inhibition of steroid synthesis can have an adjuvant action when combined with drugs that potentiate oxidative stress, such as antimony derivatives, since the combined use of these drugs increases the intracellular death of *L. donovani*.^42^ It has even been shown that antioxidant substances, such as vitamin E, are produced by protozoa, such as *P. falciparum*, to protect them from oxidative stress generated by drugs and the host’s defense mechanisms.^43^ ^,^ ^44^

In addition, studies have shown that prostaglandins are potential inducers of intracellular stress via reactive species production. J series metabolites, such as 15-Deoxy-delta^12^ ^,^ ^14^ -prostaglandin J2 (15d-PGJ_2_) and prostaglandin D2 (PGD_2_) are important bioactive lipid mediators capable of inducing an increase in reactive species in *L. donovani* and *T. brucei* promastigotes. However, further studies are needed to demonstrate LD involvement in a mechanism protecting against mitochondrial damage and death triggered by eicosanoid activation.^45^ ^,^ ^46^

Protozoan parasites LDs in the metabolism of bioactive lipids

There are still little data on the metabolism of eicosanoids in pathogenic protozoa, and most studies focus on parasites of the genera *Trypanosoma* and *Leishmania* ^24^ ^,^ ^27^ ^,^ ^33^ ^,^ ^47^ ^-^ ^53^ (Table II). A variety of specialised lipid mediators and eicosanoids have been described in protozoa, but studies on the role of these mediators in the biology of the parasites, as well as in the parasite-host interaction, are still lacking. To date, studies have demonstrated the presence of resolvins (Rvs),^48^ eicosapentaenoic acid (EPA), AA, docosahexapentaenoic acid (DHA),^33^ ^,^ ^48^ prostaglandins (PGs)^27^ ^,^ ^48^ ^,^ ^49^ ^,^ ^51^ ^,^ ^54^ ^-^ ^58^ and thromboxanes (TXs)^52^ in lipid extracts of parasites (Table II).

**TABLE II:** Lipid mediators, precursors and their metabolism enzymes identified in protozoan pathogens

Species	Lipid mediators and precursors	Proteins	References
*Leishmania* sp.	Linoneic acid-derived metabolites (9,10-DiHOME, 9,10-DiHODE, 15,16-DiHODE, 9-HODE, 10-HODE, 12-HODE, 13-HODE, 15-HODE, 5(S)-HETrE, 8(S)-HETrE, 12(S)-HETrE, 15(S)-HETrE) Arachidonic acid-derived metabolites (5-HETE, 8-HETE, 11-HETE, 12-HETE, 15-HETE, 18-HETE, 8(9)-EpETrE, PGE_2_, PGD_2_, PGF_2α_) Docosahexaenoic acid- derived metabolites (4-HDoHE, 7-HDoHE, 8-HDoHE, 10-HDoHE, 11-HDoHE, 13-HDoHE, 14-HDoHE, 16-HDoHE, 17-HDoHE, 20-HDoHE) Eicosapentaenoic acid-derived metabolites (7(8)-EpDPE, 10(11)-EpDPE, 13(14)-EpDPE, 16(17)-EpDPE, 19(20)-EpDPE, 10,11-DiHDPE, 13,14-DiHDPE, 16,17-DiHDPE)	PLA_2_/PAF-AH, PGFS, COX-like enzyme, CYP1, CYP2, CYP3	Araújo-Santos et al.^27^ Azevedo et al.^33^ Alves-Ferreira et al.^57^ Pawlowic et al.^58^ Estrada-Figueroa et al.^47^ Kabututu et al.^49^ Paloque et al.^51^
*Trypanosoma brucei*	Arachidonic acid-derived metabolites (PGE_2_, PGD_2_, PGF_2α_)	PLA_2_, PGFS	Kubata et al.^54^ ^,^ ^55^
*Trypanosoma cruzi*	Hydroxydocosahexaenoic acid precursors 17-HDHA, 14-HDHA, 7-HDHA, 4-HDHA Arachidonic acid-derived metabolites (PGE_2_ PGD_2_, PGF_2α_, TXA_2_, 5-HETE, 12-HETE, 15-HETE, 5S,15S-DiHETE, 5-HEPE, 12-HEPE, 15-HEPE, 18-HEPE, 5S,15S-DiHEPE, TXA_2_, PGE_2_, PGD_2_, PGF_2α_) Docosahexaenoic acid-derived metabolites (RvD1, RvD5) Eicosapentaenoic acid-derived metabolites (RvE2)	PGFS, PGES, TcTP, PLA_2_, TXA_2_S	Toledo et al.^24^ Colas et al.^48^ Okamoto et al.^50^ Murkherjee et al.^53^ Kubata et al.^55^ ^,^ ^56^
*Toxoplasma gondii* *Plasmodium falciparum* *Trypanosoma congolense*	Not determined	PLA_2_	Kubata et al.^55^

DiHOME: dihydroxyoctadecaenoic acid; DiHODE: dihydroxyoctadecadienoic acid; HODE: hydroxyoctadecadienoic acid; HETrE: hydroxyeicosatrienoic acid; HETE: hydroxyeicosatetraenoic acid; DiHETE: hydroxyeicosatetraenoic acid; EpETrE: epoxyeicosatrienoic acid; HDoHE: hydroxydocosahexaenoic acid; EpDPE: epoxydocosapentaenoic acid; DiHDPE: dihydroxydocosapentaenoic acid; PG: prostaglandin; TX: thromboxane; HDHA: hydroxydocosahexaenoic acid; HEPE: hydroxyeicosapentaenoic acid; DiHEPE: dihydroxyeicosapentaenoic acid; Rv: resolvin; TcTP: *T. cruzi* thromboxane receptor; PLA_2_: phospholipase A_2_; PAF-AH: platelet-activating factor-acetylhydrolase; PGFS: prostaglandin F synthase; PGES: prostaglandin E synthase; TXA2S: thromboxane A_2_ synthase; CYP: cytochrome P450 enzymes.

Parasites possess the necessary machinery for the synthesis of lipid mediators.^55^ *Leishmania* can produce PGs from PG synthases of their own LDs.^24^ ^,^ ^27^ Recently, it was discovered that the gp63 found in *Leishmania mexicana* is responsible for cyclooxygenase (COX)-like activity, emphasising the importance of eicosanoids and other lipid mediators capable of being synthesised by the parasite itself.^47^ In addition to COX, trypanosomatids have enzymes capable of synthesising other eicosanoids, such as PGE_2_, PGD_2_ and PGF_2α_ ^54^ ^,^ ^56^ *T. cruzi* trypomastigotes respond to exogenous AA stimulation with an increase in PGE synthase expression.^24^ The role of inflammatory lipids produced by the parasite during infection remains to be clarified. *L. infantum* LDs are capable of synthesising PGF_2α_, and this mediator is responsible for increasing the parasite’s viability in the initial moments of infection by a mechanism yet to be determined.^27^ Concomitantly, *T. cruzi* trypomastigotes synthesise RvD1 contributing to the resolution of the inflammatory process.^48^ *T. cruzi* is also capable of synthesising and releasing TXs, a primarily pro-inflammatory molecule, which, however, starts to show anti-inflammatory activity due to the suppression of pathways in infection *in vivo*.^52^ In this sense, the presence of a thromboxane receptor has been demonstrated in *T. cruzi*, but its role in the biology of the pathogen is still uncertain.^53^ Another pathway for the production of lipid mediators has been described in parasites.^51^ Paloque at al. described cytochrome p450-like (CYP450) proteins in the *L. infantum* genome as responsible for producing polyunsaturated FA metabolites. CYP450-like proteins from *L. infantum* seem to be responsible for lipid precursor production of specialised lipids in this parasite.^51^ Thus, *L. infantum* is capable of releasing eicosanoids and other lipid mediators, but how these mediators alter the course of infection has not been established.

Conclusions and perspectives

The lipid droplets of different pathogenic protozoa are not a simply static lipid storage sites. These organelles are dynamic sites of lipid storages, and they are especially important for the parasite to function in the context of interaction with the cell host. LDs display a central importance in the growth, differentiation, infectivity, and lipid metabolism of protozoan parasites (Table I). Proteomic and lipidomic studies on isolation of protozoan lipid droplets are still needed to establish the functions performed by these organelles. Furthermore, comparative genomic studies can be conducted to transpose the knowledge of different pathogenic protozoa to identify the genes responsible for the biogenesis and elimination of LDs. An understanding of lipid metabolism, especially of bioactive lipids, and the role of protozoan LDs in this context, may help to identify potential targets for the development of antiparasitic drugs. Therefore, LDs are promising research targets for future methods of controlling infection based on the cell biology of pathogenic protozoa.
